# Brain Magnetic Resonance Imaging-Based Evaluation of Pediatric Patients With Developmental Delay: A Cross-Sectional Study

**DOI:** 10.7759/cureus.24051

**Published:** 2022-04-11

**Authors:** Harneet S Randhawa, Sachin Bagale, Rajesh Umap, Jasneet Randhawa

**Affiliations:** 1 Radiology, Sassoon General Hospital, Pune, IND; 2 Radiology, Government Medical College, Baramati, Pune, IND; 3 Radiology, Aulakh Hospital, Amritsar, IND; 4 Radiology, Fortis Escorts Hospital Amritsar, Amritsar, IND

**Keywords:** global developmental delay, perinatal asphyxia, developmental delay, pediatrics, brain

## Abstract

Background

Developmental delay refers to the insufficient acquisition of age-appropriate developmental milestones. According to World Health Organization, approximately 5% of all children under the age of 14 years display some developmental disability.

Aim and objective

Our objective was to investigate the prevalence of abnormal magnetic resonance imaging (MRI) brain findings in pediatric patients with non-syndromic developmental delay and to establish the utility of MRI for the same.

Material and Method

This cross-sectional study prospectively enrolled 60 pediatric patients (three months to 12 years) and data were analyzed using SPSS software.

Result

Abnormalities on MRI were seen in 80% of cases, with findings indicating perinatal hypoxic insult (36.67%) being the most common, followed by structural abnormalities of the brain (20%). There was no significant difference in the prevalence of abnormal findings when classified by gender or age, or between global developmental delay (GDD) alone and GDD with epilepsy. However, perinatal hypoxic insult was significantly associated with GDD with epilepsy rather than GDD alone (p < 0.01).

Conclusion

In this study, brain MRI provides a high yield of abnormal findings and helps calculate the relative prevalence of various common etiologies in non-syndromic developmental delay. This study supports several international guidelines that include MRI as the first-line investigation for non-syndromic developmental delay.

## Introduction

Developmental delay is diagnosed when a child shows a significant lag (2 standard deviations below the mean in one domain or 1.5 standard deviations in any two domains) in the acquisition of age-appropriate developmental milestones. The relevant domains of development evaluated include gross motor function, fine motor function, speech/language, personal/social milestones, and cognition. Global developmental delay (GDD) refers to under-achievement in two or more domains, whereas insufficient progress in a single domain is termed a specific developmental delay (SDD) [1].

Although comprehensive data for the Indian population is not available, studies from other countries have shown some developmental disability in about 15% of children, and according to WHO estimates of the global burden of disease, approximately 5% of all children under the age of 14 years display some developmental disability. Additionally, GDD alone has been reported in 1%-3% of the pediatric population aged five years or younger [1-3].

Known etiologies of developmental delay include perinatal hypoxic insult, structural brain abnormalities, metabolic defects, toxins, infections, genetic syndromes, and environmental factors, and while patients with developmental delay are subjected to detailed history and physical examination, and chromosomal and metabolic studies, MRI is typically used only as a second-line modality [4]. Thus, the aim of our study was to ascertain the prevalence of abnormal MRI brain findings in Indian pediatric patients with developmental delay and to categorize those findings, apart from assessing the utility of brain MRI in evaluating these patients.

## Materials and methods

Study design

This cross-sectional study on brain MRI findings was performed over a period of 18 months, at the Radiology Department in our hospital.

Inclusion criteria

Pediatric patients with clinically diagnosed developmental delay who were between three months and 12 years old and had been referred to our department for brain MRI were included in the study.

Exclusion criteria

1) Patients below three months or above 12 years of age; 2) patients with genetic disorders, like Down syndrome where chromosomal investigations may be sufficient for establishing the cause of the developmental delay; and 3) patients too sick to undergo MRI or in whom MRI is contraindicated.

Sample

A non-random sample that included all pediatric patients with developmental delay who underwent brain MRI and met the inclusion and exclusion criteria.

Materials

Brain MRI scans were acquired on a 1.5 Tesla GE Signa HDxt machine with a radiofrequency head coil. The field of view was kept as small as possible and a 256 x 256 matrix was used. Slice thickness was 3-5 mm and the interslice gap was 1 mm. Axial T1 and T2, GRE, DWI (with ADC), Axial T1 and T2 FLAIR, sagittal T1, and coronal T2 sequences were acquired. Gadolinium-based contrast medium was used only when indicated and patients were sedated by a trained anesthesiologist is required.

Methodology

The study protocol was approved by the institutional ethics committee. During the study period, 65 patients were referred for a brain MRI to ascertain the cause of the developmental delay; of these, two had Down syndrome and three were too ill to be sedated for a full scan, and hence, were not included in the study. Details of the study were explained to the parents/guardians of the remaining 60 patients in their vernacular language and patients were included in the study only after written informed consent was provided.

MRI scans were evaluated in detail and findings were divided into the following groups:

1. Perinatal hypoxic insult: based on the clinical history and MRI abnormalities; this group included both mild and severe hypoxic insult in the perinatal period in preterm/term gestation children. 

2. Structural brain abnormalities

3. White matter/metabolic disease

4. Other findings, such as abnormal signal intensities and nonspecific findings which could not be included in the above categories

5. Normal MRI

Statistical analysis

Data are presented as normal and abnormal MRI where abnormal findings were further categorized into a perinatal hypoxic insult, structural brain abnormalities, white matter/metabolic disease, and other findings. Total number and percentage prevalence were calculated and studied for differences in prevalence among age, gender, mode of delivery, and clinical presentation. Data were tabulated and analyzed using the Chi-squared test. All analyses were performed on SPSS software (ver Build 1.0.0.1447, 64-bit edition), and a p-value of <0.05 was considered statistically significant.

Bias

Since all the patients included in the study sample were referred for scanning and met the inclusion criteria, there is unavoidable selection bias.

## Results

The study population had most patients (21) aged ˂2 years with a higher number of male patients (33). Most of the patients were born via vaginal delivery. Clinically most of them were diagnosed with GDD followed by GDD with epilepsy. All patients in this study had findings that could be categorized into only one group and no overlap was noted. The most common MRI abnormality was a hypoxic insult (36.67%), followed by structural brain abnormalities (20%) and other findings (18.33%). Only a few cases showed findings consistent with white matter/metabolic diseases (5%). No obvious confounding factors were identified in our study (Tables 1, 2).

**Table 1:** Characteristics of the study population

	Total no. of patients in each category	Abnormal findings in MRI (number of patients)
Gender		
Male	33	25
Female	27	23
Age (years)		
<2	21	17
2–5	15	12
6–10	16	12
>10	8	7
Mode of delivery		
Vaginal delivery	53	43
Caesarean section	7	5
Clinical Diagnosis		
Global developmental delay only	39	31
Specific developmental delay	3	2
Global developmental delay plus convulsions	18	15

 

**Table 2:** MRI findings of the patients enrolled in the study

Finding	Number of patients	Percentage
Perinatal hypoxic insult	22	36.67
Structural brain abnormality	12	20
Metabolic/White matter abnormality	3	5
Others	11	18.33
Normal MRI	12	20
Total	60	100

Chi-squared test for normal and abnormal brain MRI findings in male and female patients showed no significant difference (p = 0.364). A comparison of the prevalence of significant brain MRI findings in children younger than two years with those in other groups resulted in p-values of 0.943, 0.663 and 0.677 for <2 y vs.2-5 y, <2 y vs. 6-10 y and <2 y vs. >10 y, respectively. Further, a comparison of the prevalence of abnormal brain MRI findings in patients born by vaginal delivery and caesarean section had a p-value of 0.546. Taken together, these results imply that no significant differences were noted in prevalence of abnormal brain MRI findings among different age groups, gender or mode of delivery.

The prevalence of abnormal brain MRI findings in GDD and GDD with epilepsy was similar (p = 0.732). In contrast, when perinatal insult as a cause of developmental delay was compared between GDD alone and GDD with epilepsy, we obtained a p-value of 0.01, suggesting that perinatal hypoxic insult may be more commonly associated with GDD with epilepsy rather than GDD alone. Next, the most common structural brain abnormalities in our study were those of the corpus callosum and neuronal migration defects. Structural brain abnormalities were comparable between GDD alone and GDD with epilepsy (p = 0.211), and similarly no significant difference was found when metabolic/white matter disease (p = 0.946) or other parameters (p = 0.106) were tested as potential causes of GDD or GDD with epilepsy (Tables 3-6).

**Table 3:** MRI findings in global developmental delay alone

Finding	Number of patients	Percentage
Perinatal hypoxic insult	10	25.6
Structural Brain abnormality	10	25.6
Metabolic/White matter abnormality	2	5.2
Others	9	23.1
Normal MRI	8	20.5
Total	39	100

**Table 4:** MRI findings in global developmental delay with epilepsy

Finding	Number of patients	Percentage
Perinatal hypoxic insult	11	61.11
Structural Brain abnormality	2	11.11
Metabolic/White matter abnormality	1	5.55
Others	1	5.56
Normal MRI	3	16.67
Total	18	100

 

**Table 5:** Structural brain abnormalities

Finding	Number	Percentage
Corpus callosum agenesis	1	8.33
Corpus callosum hypoplasia	2	16.67
Pachygyria	2	16.67
Heterotropia	1	8.33
Mega cisterna magna	2	16.67
Joubert syndrome	1	8.33
Cerebellar atrophy	1	8.33
Asymmetric ventricles	2	16.67
Total	12	100

**Table 6:** MRI findings in specific developmental delay

Finding	Number	Percentage
Perinatal hypoxic insult	1	33.33
Others	1	33.33
Normal MRI	1	33.33

## Discussion

Comprehensive data on brain MRI findings in developmental delay are not available for the pediatric Indian population, and therefore, the objective of our study was to estimate the prevalence of abnormal MRI findings and categorize them, and subsequently use these findings to establish the utility of MRI in such patients. Our cross-sectional study included 60 patients aged between three months and 12 years who were clinically diagnosed with developmental delay.

This study shows that the prevalence of abnormal brain MRI findings was high at 80% and similar prevalence rates have been reported in the studies by Momen et al. (58.6%), Pandey et al. (68.3%), Shevell et al. (65.5%), Ali et al. (68%), Koul et al. (71.8%), Battaglia et al. (80.8%), and Widjaja et al. (84%) [2,5-10].

The study population had more male patients compared to females (1.22:1), and while other studies have also reported a higher proportion of males, e.g., Momen et al. (1.3:1) and Kaur et al. (2.4:1), in this study no significant difference in the prevalence of abnormal MRI findings between male and female patients (p = 0.364). Similar results have been described in the study by Ali et al. (Table 1) [2,5,11]. 

Most patients in the study were under two years of age but no significant difference in the prevalence of abnormal brain MRI findings among the different age groups were noted; these results concur with those reported in the study by Ali et al. (Table 1) [5].

More patients were born by normal vaginal delivery rather than cesarean section, but no significant difference in the prevalence of abnormal brain MRI findings was noted between the two groups. Nevertheless, some studies have suggested that a planned cesarean section carries a lower risk of birth complications compared to a planned vaginal delivery (Table 1) [12,13]. 

**Figure 1:**
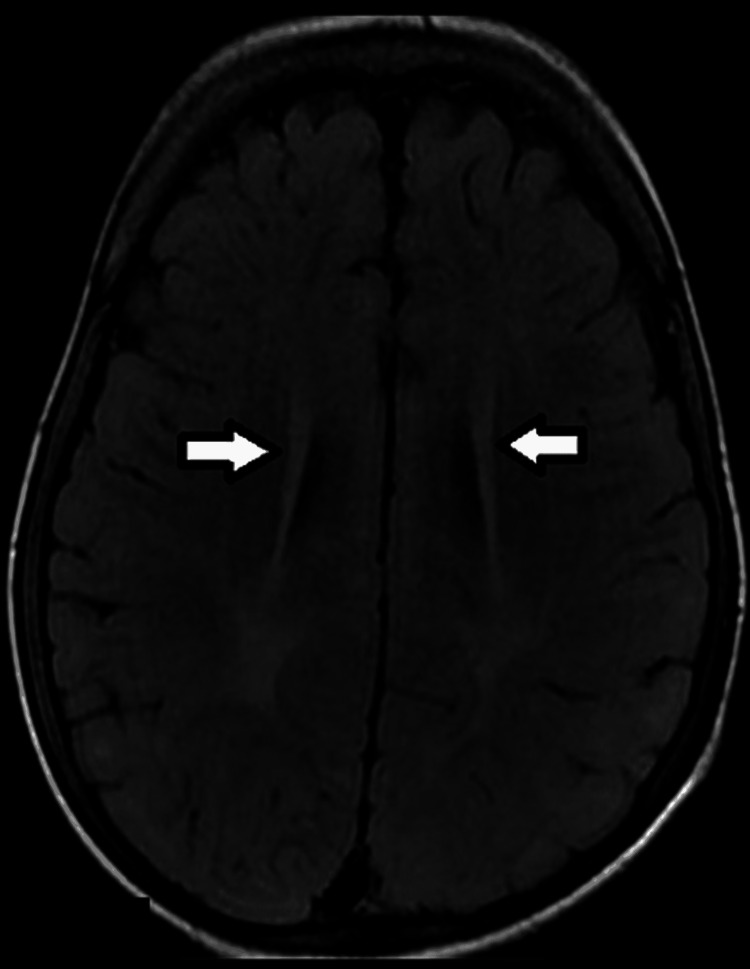
Axial T2 FLAIR image in a 18-month male showing an abnormal hyperintense signal in bilateral periventricular region, consistent with changes of periventricular leukomalacia (white arrows).

**Figure 2:**
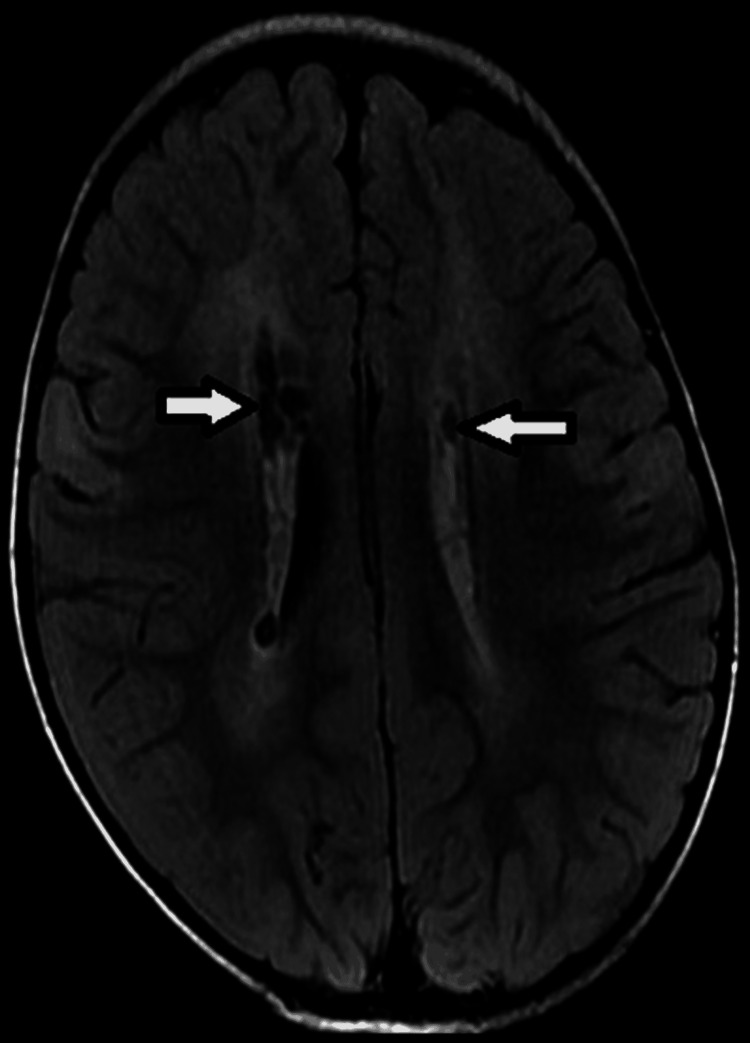
Axial T2 FLAIR images of a three-year-old child with developmental delay, at the level of the Corona radiate, showing periventricular cystic encephalomalacia and adjacent gliosis due to perinatal hypoxic insult (white arrows).

**Figure 3:**
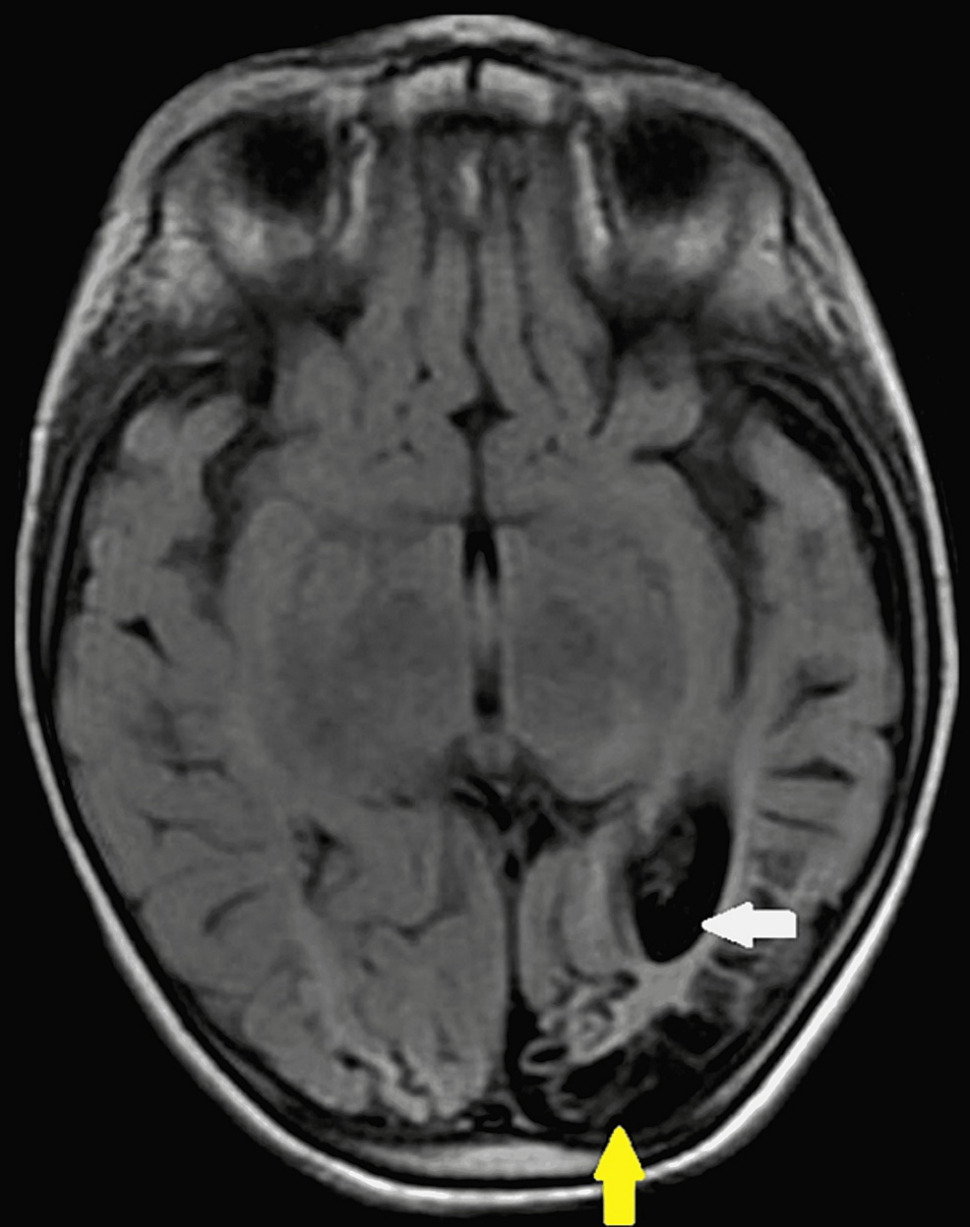
Axial T2 FLAIR image of a two-year-old child with developmental delay showing cystic encephalomalacia with adjacent gliosis (yellow arrow), volume loss, and ex-vacuo dilatation of the  occipital horn of the left lateral ventricle (white arrow).

**Figure 4:**
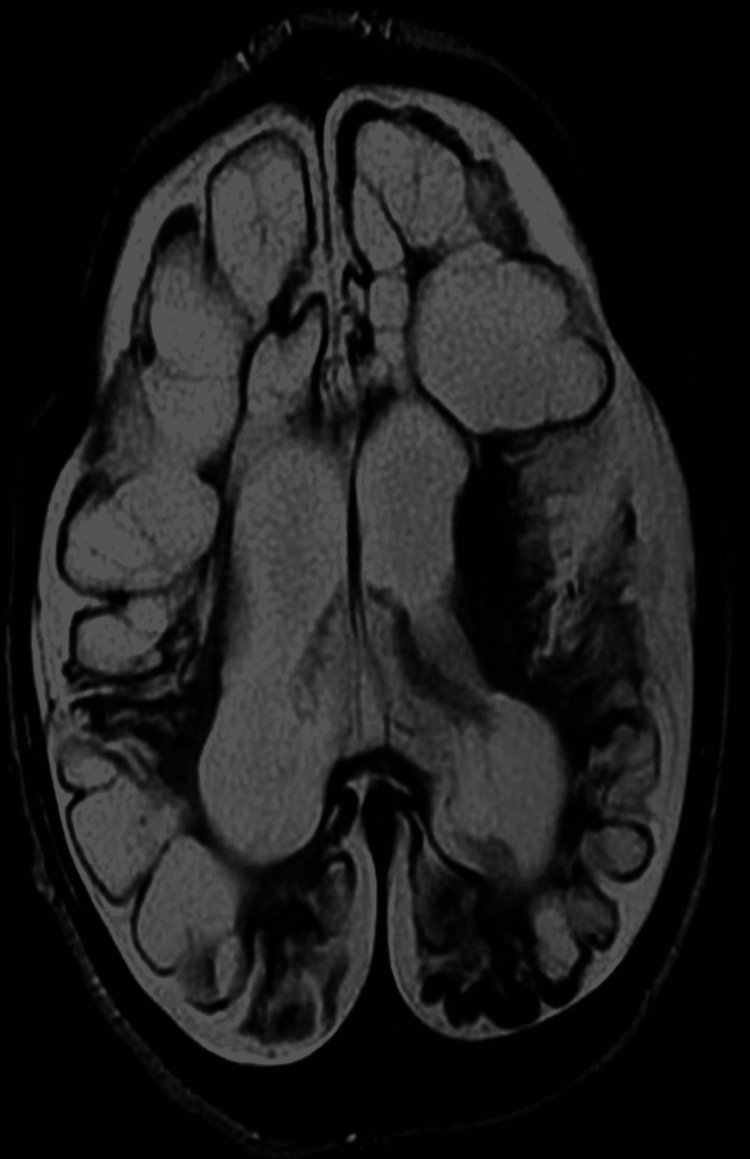
Axial T2W images of an eight-month-old child with severe perinatal hypoxic insult showing extensive encephalomacic changes with marked loss of white matter and ex vacuo ventricular dilatation.

**Figure 5:**
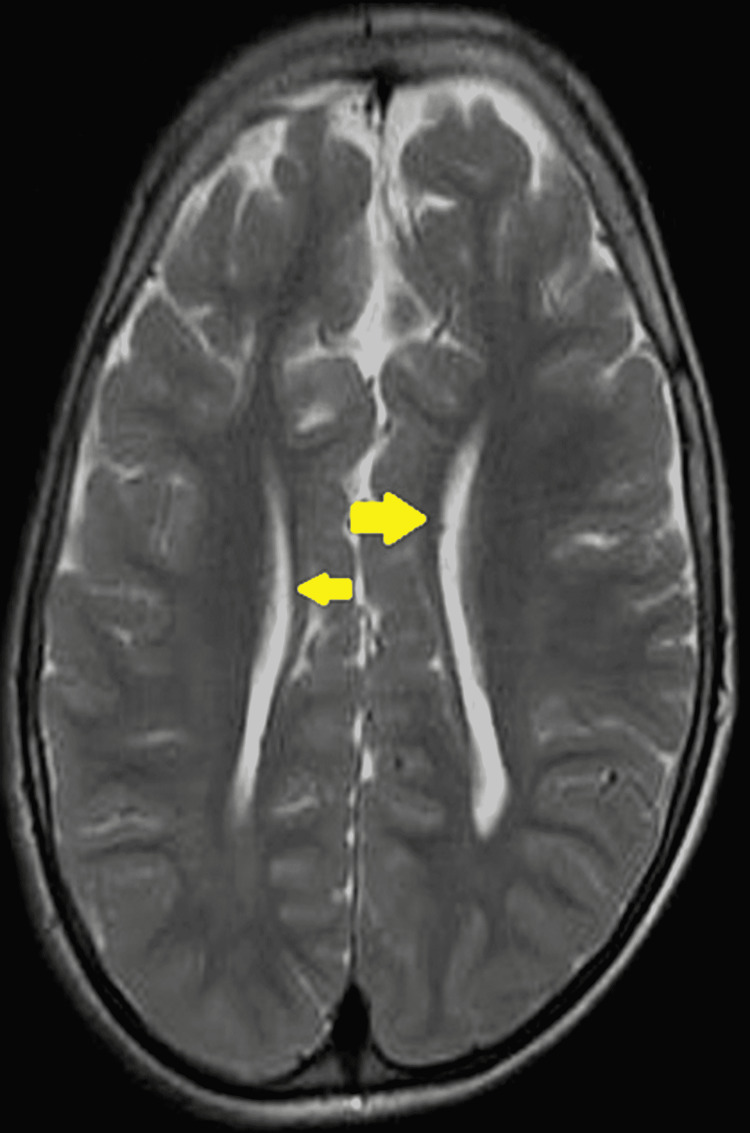
T2W axial image showing parallelly oriented lateral ventricles with an uncrossed Probst bundle (yellow arrows) adjacent to it in a patient with corpus callosum agenesis.

**Figure 6:**
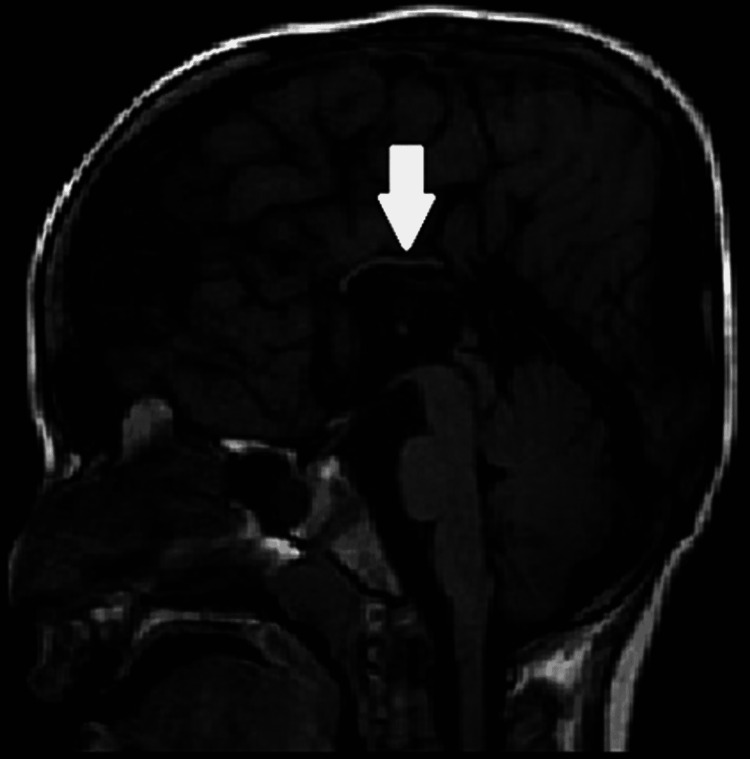
Mid-sagittal axial T1 image of a two-year-old child with developmental delay showing markedly hypoplastic corpus callosum (arrow).

**Figure 7:**
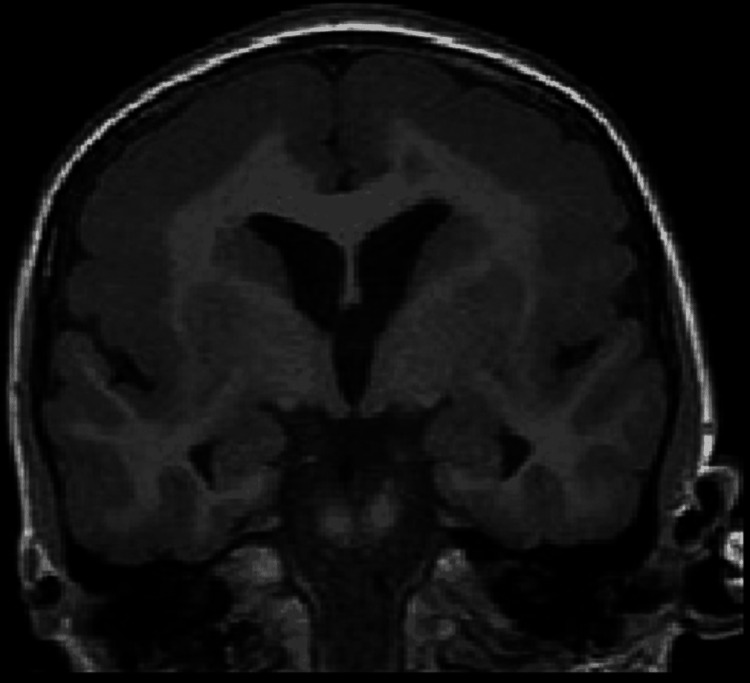
Coronal T1W image in a four-year-old male child with developmental delay shows markedly thickened grey matter with few and shallow sulci in the bilateral frontal region. The findings are consistent with pachygyria. A focus on heterotropic grey matter is also noted in the left frontal region.

**Figure 8:**
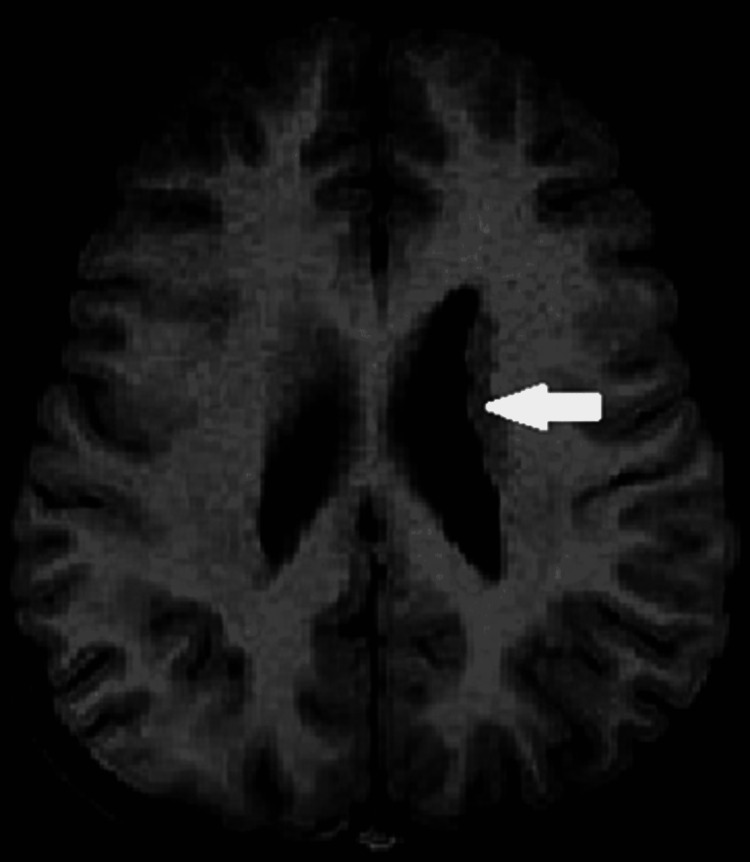
Axial T1W image of a six-year-old female child with developmental delay showing nodular grey matter intensities in the subependymal region consistent with nodular subependymal heterotropia (arrow).

**Figure 9:**
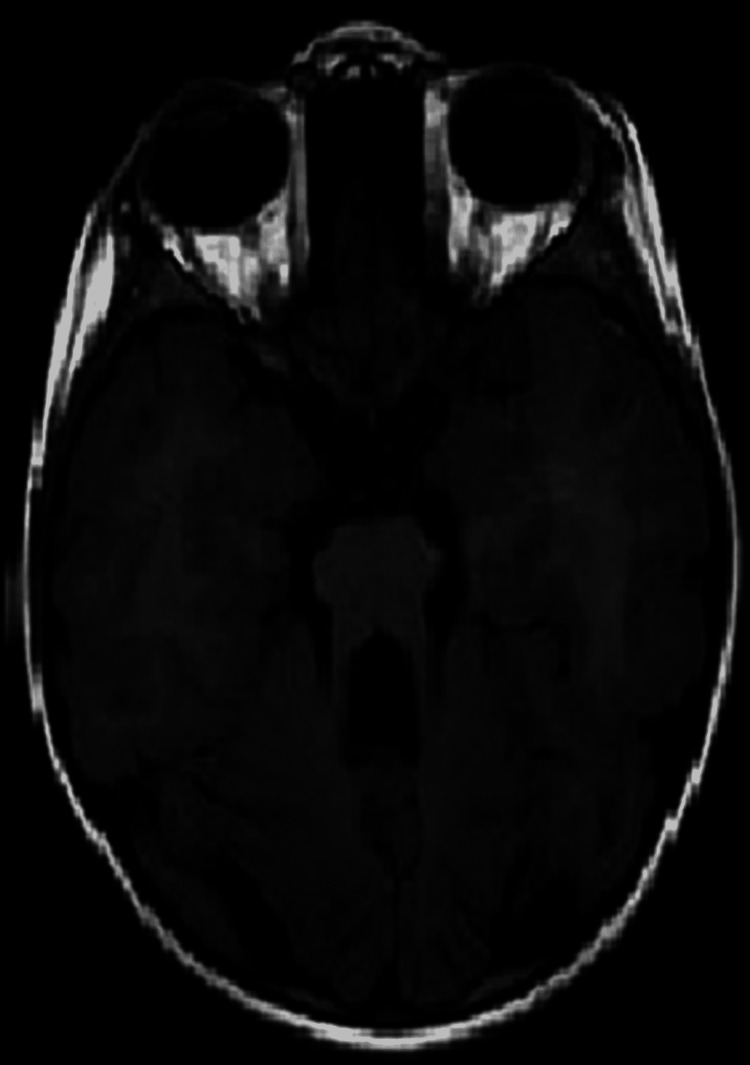
Axial T1W image of a six-year-old child with developmental delay showing prominent thickened and elongated cerebellar peduncles giving molar tooth appearance in a patient with Joubert syndrome.

In this study, the most common MRI abnormality was hypoxic insult (Table 2). Studies by Ali et al. (31%) and Koul et al. (23.7%) have also shown similar results. However, a congenital structural abnormality was the second most common finding in a study by Ali et al. (17%), whereas “other findings” was the second most common in the study by Koul et al. (25.2%). This difference in the results from the Koul et al. study may be due to the fact that the selection criteria were different (Figures 1-9) [5,8]. 

Most patients in the study were clinically diagnosed with GDD followed by the global developmental delay with convulsions (GDD + convulsions), and only three patients had SDD. No significant difference was noted in the prevalence of abnormal brain MRI findings between GDD and GDD with convulsions. However, a study by Schaefer et al. reported that the yield of abnormal findings was higher when convulsions or other clinical findings are present along with GDD (Tables 1, 3-5) [14].

The study demonstrates that perinatal hypoxic insult was the most common brain MRI finding in all groups and that it was significantly associated with GDD with convulsions rather than GDD alone. This study shows no significant difference in other etiologies associated with GDD or GDD with convulsions; however, this was a cross-sectional study and hence, longitudinal studies are required to better assess the risk of developmental delay due to these etiologies. Additionally, the number of patients in the SDD group was too small for a significant comparison of etiologies (Tables 3, 4).

Limitations

DTI, tractography, and MR spectroscopy could not be performed even though many studies have shown that these can further increase the yield of abnormal MRI findings [15,16]. Another limitation is the non-availability of similar data from normal pediatric patients (i.e., without developmental delay) for comparison. Lastly, as the study was conducted at a tertiary health care center with non-random sampling that included all eligible patients, there may be some selection bias, and this data may not accurately represent the prevalence of abnormal brain MRI findings in the general population as peripheral areas do not have enough access to advanced services like MRI.

## Conclusions

The developmental delay has multiple etiologies, many of which cannot be diagnosed without the use of neuroimaging, such as the degree of perinatal hypoxic insult and structural brain abnormalities. In this study, brain MRI provides a high yield of abnormal findings and helps calculate the relative prevalence of various common etiologies in non-syndromic developmental delay. This study supports several international guidelines that include MRI as the first-line investigation for non-syndromic developmental delay. Future longitudinal studies are required to further strengthen this evidence.
